# Systematic Review Protocol to Assess the Effectiveness of Usability Questionnaires in mHealth App Studies

**DOI:** 10.2196/resprot.7826

**Published:** 2017-08-01

**Authors:** Leming Zhou, Jie Bao, Bambang Parmanto

**Affiliations:** ^1^ University of Pittsburgh Department of Health Information Management Pittsburgh, PA United States

**Keywords:** usability, questionnaire, mobile health app, systematic review

## Abstract

**Background:**

Usability questionnaires have a wide use in mobile health (mHealth) app usability studies. However, no systematic review has been conducted for assessing the effectiveness of these questionnaires.

**Objective:**

This paper describes a protocol for conducting a systematic review of published questionnaire-based mHealth app usability studies.

**Methods:**

In this systematic review, we will select recently published (2008-2017) articles from peer-reviewed journals and conferences that describe mHealth app usability studies and implement at least one usability questionnaire. The search strategy will include terms such as “mobile app” and “usability.” Multiple databases such as PubMed, CINAHL, IEEE Xplore, ACM Digital Library, and INSPEC will be searched. There will be 2 independent reviewers in charge of screening titles and abstracts as well as determining those articles that should be included for a full-text review. The third reviewer will act as a mediator between the other 2 reviewers. Moreover, a data extraction form will be created and used during the full article data analysis. Notably, the Preferred Reporting Items for Systematic Review and Meta-Analysis Protocols (PRISMA-P) guidelines will be followed in reporting this protocol.

**Results:**

A preliminary search produced 1271 articles, 40 of which are duplicate records. The inclusion-exclusion criteria are being strictly followed in performing the ongoing study selection.

**Conclusions:**

Usability questionnaires are an important tool in mHealth app usability studies. This review will summarize the usability questionnaires used in published research articles while assessing the efficacy of these questionnaires in determining the usability of mHealth apps.

## Introduction

In recent years, a large number of mobile health (mHealth) apps have been created to augment various personal health regimens including weight loss, smoking cessation, chronic disease management, virtual clinical visits, and medical education. These apps have been evaluated in various usability studies—a critical step in determining the quality of the apps.

According to the International Organization for Standardization, usability marks “the extent to which a product can be used by specified users to achieve specified goals with effectiveness, efficiency, and satisfaction in a specified context of use” [1]. This definition provides one usability model: effectiveness, efficiency, and satisfaction. Another widely cited usability study model included five alternative components: efficiency, satisfaction, learnability, memorability, and errors [2]. Additionally, there are existing efforts for creating new usability models specifically for mobile apps by considering new usability challenges (eg, mobility, connectivity, and additional cognitive load) introduced by mobile devices [3]. However, without a thorough evaluation, it is hard to determine which usability model is the best for mHealth app usability studies.

There are many usability study methods. These methods can be categorized into two major types according to the usability study participants. In the first method style, participants are the members of a research/development team or a group of usability study experts. In these roles, they perform cognitive walkthroughs or heuristic evaluations of the mHealth app to determine the app’s usability [4]. In the second method style, participants are selected from a pool of the app’s actual users. Typically, these participants are required to use the app to finish a number of tasks and then provide their feedback. Here, not only are the participants’ performances and activities logged and analyzed, but their opinions of the product are also collected using study questionnaires, focus groups, or interviews. These participants may be encouraged to speak out when working on their tasks to tell researchers their ideas or comments (think aloud).

There are currently a number of validated and reliable usability questionnaires such as the Post-Study System Usability Questionnaire (PSSUQ) [5], System Usability Scale (SUS) [6], Usefulness, Satisfaction, and Ease of Use Questionnaire [7], Telehealth Usability Questionnaire [8], Questionnaire for User Interface Satisfaction [9], Perceived Usefulness and Ease of Use [10], and the Health Information Technology Usability Evaluation Scale [11], to name just a few. These questionnaires (especially SUS and PSSUQ) have been used in a number of mHealth app usability studies because of their previous wide use in IT system usability studies. None of these validated and widely used questionnaires, however, were specifically designed for evaluating the usability of individual mHealth apps, and there is no specific study on the consistency of usability study results from these questionnaires and other usability study methods. Note that there are a number of studies on mHealth app usability evaluation for a group of apps, with the purpose of selecting the best app for some specific tasks such as chronic disease management, headache diary, weight loss, and smoking cessation [12-15]. Because of the significant difference in the research purposes, these studies are substantially different from this study. However, the results obtained in these studies may be useful in this usability questionnaire effectiveness study; therefore, they will be assessed in this study.

mHealth apps have many inherent characteristics limiting their usability. For instance, mHealth apps typically run on mobile devices such as phones and tablets. Mobile phones have a small screen size, tiny fonts, and soft keyboards, which may limit the interactions between the user and the app [3]. For health care purposes, mHealth apps need to be on and available at all times, otherwise users could find themselves in a life-threatening situation. Furthermore, since mHealth apps often need to handle real-time and highly sensitive communications between patients and health care providers, existing usability questionnaires used in the mHealth app usability studies must be sufficiently evaluated so as to determine which ones are the most effective in assessing mHealth app’s usability.

There are several review articles [3,16,17] published regarding usability studies in mobile apps. Most of these reviews, however, did not focus specifically on the evaluation of the usability questionnaires used in mHealth app usability studies or they did not compare the result consistency between the usability questionnaires and other usability study methods. Therefore, this systematic review protocol aims at describing the procedure underlying the design of a systematic review for evaluating the effectiveness of usability questionnaires implemented in published mHealth apps. Here, the “effectiveness” indicates that the usability questionnaires can obtain similar results in terms of the app’s usability as obtained from other usability study methods. In other words, high effectiveness indicates that the results from the usability questionnaires are highly consistent with the results from other usability study methods on usability aspects they both can measure. The results of this systematic review may lead to the creation of usability questionnaire selection guidelines for researchers seeking to evaluate the usability of mHealth apps using questionnaires.

## Methods

The construction of this systematic protocol followed Preferred Reporting Items for Systematic Reviews and Meta-Analyses Protocol (PRISMA-P) guidelines [18], which recommend a number of essential components in systematic reviews. Those components most relevant to this review study will be described in detail later.

### Search Strategies

The search strategy consists of keywords appropriate to the objective of the review. More specifically, in the initial literature query, our search strategy will simply entail “mobile app” AND “usability.” Notably, this initial query contains no keywords relating to health, since there are many different ways of describing health-related mobile apps. Searches will first be performed in bibliographic databases PubMed, CINAHL, INSPEC, ACM Digital Library, and IEEE Xplore digital library. These databases include a huge number of journal articles and conference proceedings. From here, the obtained studies will be selected by following the inclusion and exclusion criteria described below. All reference lists of selected articles will be evaluated, and the studies meeting the inclusion criteria will be added into the review list. Searches on other suitable resources will also be performed if that the search in the five previously listed databases does not produce a sufficient number of studies. Other suitable resources include conference proceedings and reports that are not indexed by the five databases. If necessary, study authors will be contacted if they have mentioned other relevant studies without delineation.

### Inclusion and Exclusion Criteria

In general, studies on mobile app usability will be included in the initial results if they were both published in English and in peer-reviewed journals or conferences. More specific inclusion and exclusion criteria related to time frame, participants, types of studies, interventions, and outcomes are described below.

All studies published between January 2008 and March 2017 will be included in the review. It is believed that the database search will produce only a small number of studies published before 2008. This can be explained by considering the history of mobile phone app development. Prior to 2008, although there were some mobile health apps, they were typically text messaging‒based apps. The major concentration of those studies was on the content of messages and timing or frequency of delivering those messages. Therefore, the usability of those mobile apps was typically not extensively evaluated or reported.

The participants of usability studies should include targeted users of the designed app in the study instead of relying solely on the research team or usability evaluation experts. The selected studies should include usability evaluation with at least two methods—cognitive walkthrough, think aloud, questionnaires, log and usage file analysis, and heuristic evaluation done by usability experts. One of the methods must be a usability questionnaire answered by a group of targeted app users.

Studies that examined the usability of individual mHealth apps using one or more usability questionnaires will be included in this review. Such studies might include randomized and nonrandomized controlled trials, pre/posttest designs, nonexperimental observation (cross-sectional, case series, case studies), and qualitative studies. Studies will be excluded if they are not about mobile apps, not related to health, not usability studies, or did not use any questionnaires in the usability study. Additionally, studies will be excluded if they are editorials, letters to the editor, interviews, study protocols, reviews, position papers, and opinion papers. Furthermore, if the number of participants in a given usability study is extremely small (<4) or the questionnaire is very brief (<4 questions in the entire questionnaire, including subquestions), the corresponding studies will also be excluded from this review.

There is no restriction on the intervention but the intervention must be delivered by or through the mobile apps, for instance, generating reminders, providing education materials, collecting data from users actively, and assisting with the communications between users and other parties (eg, patients vs health care providers, patients vs caregivers, users and their friends). In other words, the users had interactions with the mHealth apps via the interface of the apps. Studies will be excluded if the described mHealth apps collect data from the users only silently and the users never need to interact with the app.

The outcomes will be collected from those studies in this systematic review that contain usability questionnaires. Note that in this review we will not evaluate the quality of those apps, but rather we will evaluate the consistency between the usability study results from the chosen questionnaires and the results from other usability study methods. Both results should be reported in the selected studies. If a particular study does not yield either of these outcomes, it will be removed from this systematic review.

### Study Record Management

All search results will be exported into an EndNote library (Clarivate Analytics). The citations from all searches will be evaluated, and duplicate records will be removed using EndNote. The portable document format (PDF) files of all reviewed articles are to be stored in a shared Box folder (Box, Inc.). Additionally, study selection results, research team discussion notes, and data extraction forms will be stored in this shared Box folder as well.

In certain cases, duplicated publications (with different titles and authors) may be encountered during the study selection. The authors and those studies will be carefully compared and evaluated. If they show no significant difference, the duplicated studies will be removed from the study records. When needed, study authors may be contacted to clarify whether the same study participants, questionnaires, and mobile apps were used in multiple studies.

### Study Selection

In the first round of the study selection, 2 reviewers (LZ and JB) will independently select studies by reading the titles and abstracts of all obtained studies and determining their eligibility based on the inclusion/exclusion criteria. A third reviewer (BP) will resolve any disagreements between LZ and JB. All three reviewers will reach an agreement on all selected studies.

After both reviewers (LZ and JB) finish their study selections, interrater reliability will be measured using Cohen’s kappa [19]. To achieve a high kappa score, reviewers should foster extensive discussion regarding the inclusion/exclusion criteria before performing the study selection. Reviewers may also make study selections on a small set of the database search results so as to determine whether or not they apply the inclusion/exclusion criteria in the same way.

In the second round of the study selection, full-text articles of the studies selected in the first round will be downloaded and screened according to the inclusion/exclusion criteria. Each reviewer will work on one third of these studies and the selection results will be combined for further discussion among the 3 reviewers. The 3 reviewers should reach consensus on all selected studies in the second round since these studies will be included in the systematic review. The consensus will be reached by having extensive discussions among 3 reviewers.

Furthermore, the 3 reviewers will screen the articles in the reference lists of the selected studies from the second round using an identical procedure. Some articles in the reference list may be selected and added into the systematic review.

**Figure 1:**
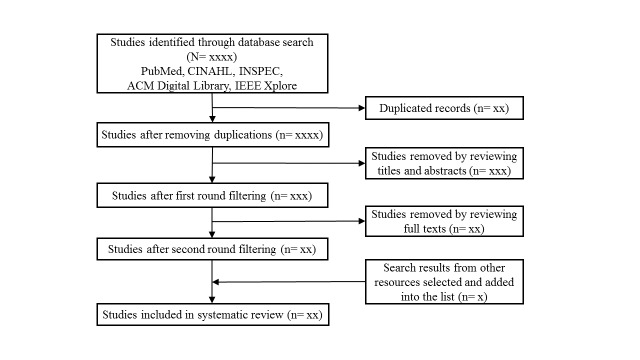
Flow diagram illustrating the study selection process.

If the number of studies identified is small after the completion of the previous step (<10), other databases and websites may be searched to identify highly relevant studies that are not indexed by the five databases. In most cases, this step is not necessary. The entire study selection procedure is demonstrated in Figure 1.

### Data Extraction

Each study selected in the previous step will be reviewed and the information about the study will be extracted and documented using a data extraction form by the search team (LZ, JB, and BP). In most cases, the full text of the study and the supplementary materials are sufficient for the purpose of data extraction. Sometimes study authors may need to be contacted if they describe the usability study results but do not provide the usability questionnaire itself.

The data extraction form captures the following data items: paper information, app information, descriptions of usability study methods, study participants, questionnaires, and usability study results. The study participants’ characteristics, their performance in the usability study, their answers to questions in the questionnaire, and their comments made during the study will be collected. If needed, further details will be requested from authors of some studies.

After the data extraction has been completed, the quality of studies and publication bias will be evaluated. Publication bias can be determined by using the Egger’s test paired with a funnel plot [20]. The quality of the study will be assessed using appropriate tools such as the Cochrane Collaboration’s Risk of Bias tool for randomized controlled trials [21,22] and the Grading of Recommendations, Assessment, Development and Evaluation (GRADE) system [23,24]. Other observational studies will be assessed using the Newcastle-Ottawa quality assessment scale [25].

### Data Synthesis

First, the study results will be narratively summarized and synthesized. This narrative synthesis will include the types of mHealth apps, the summary of each questionnaire, the usability models applied in each study, the specific measured properties (eg, usefulness, learnability, satisfaction), and other usability study methods. Then, the consistency between the results from questionnaire-based usability studies and the ones from other usability study methods will be determined. For instance, in a usability study, study participants are usually asked whether or not the app is easy to use. Moreover, all their activities on the app can be recorded, including individual button clicks, entered data, and finger movement. These two pieces of data can be compared to determine the consistency. Consider the case where one study participant chose “strongly agree” on the ease of use statement in a usability questionnaire but actually took a long time or a lot of efforts to have one simple task done. The consistency between these two usability study methods would not be high. Notably, the comparison and analysis will be qualitative since usability study results are highly subjective and are often presented as opinions or comments. Summary measurements may include descriptive statistics such as frequencies, percentages, measures of central tendency, and variation.

If subgroups are available in the studies—that is, the same type of apps and similar usability studies—more in-depth comparisons may be performed within these subgroups. This comparison result will remove the contribution from mHealth app types and the usability study methods and therefore better reflect the result differences from different usability study methods. Moreover, comparative content analysis may be employed to determine themes across qualitative data by using NVivo software (QSR International).

## Results

This study aims to determine the effectiveness of questionnaire-based usability studies in evaluating mHealth app usability as a comparison to other usability study methods commonly used in mobile app evaluation. Currently, searches in the five selected databases have been performed and 1271 studies have been identified. We have removed 40 duplicated records in these citations. The first round study selection is ongoing. It is expected that the project will be completed in 2017. The results of this study will be used to determine which questionnaire is the most effective in determining the usability of mHealth apps and whether it is necessary to create a new usability questionnaire specifically for mHealth apps.

It is anticipated that some selected articles will have reported only an overall summary of their usability studies, or complementary usability components from different study methods. In this case, authors will be directly contacted to provide more detailed data. If these authors do not respond to the request, these articles shall either be removed from this review study or only used to conduct a comparison for available usability study results.

## Discussion

### Principal Considerations

Questionnaires have been widely used in mHealth app usability studies. There is no specific guideline, however, on the selection of questionnaires in mHealth app usability studies. Often, researchers simply choose a well-known usability questionnaire such as PSSUQ and SUS [5,6] or decide to create their own questionnaires. Questionnaires such as PSSUQ and SUS were not designed for mHealth apps and many newly created usability questionnaires were for only one specific app or were not validated at all. Even so, it is believed that these questionnaires were still useful in determining the usability of mHealth apps.

This systematic review will collect these studies and perform a systematic evaluation on the effectiveness of each questionnaire in usability studies. This in turn may help researchers to make proper selections when choosing a usability questionnaire for their mHealth studies.

### Conclusion

This systematic review protocol is used to demonstrate the transparency of this review study and also provide other investigators with a methodology to conduct systematic reviews.

## Abbreviations

mHealth: mobile health
PRISMA-P: Preferred Reporting Items for Systematic Review and Meta-Analysis Protocols
PSSUQ: Post-Study System Usability Questionnaire
SUS: System Usability Scale
